# COVID-19 breakthrough infections during the circulation of Delta and Omicron variants in Tirana, Albania, April 2021**–**March 2022

**DOI:** 10.1371/journal.pone.0335197

**Published:** 2025-10-30

**Authors:** Fiona Konomi, Silvia Bino, Kujtim Mersini, Arlinda Ramaj, Kostas Danis

**Affiliations:** 1 Department of Epidemiology and Environmental Health, Local Public Health Care Directorate, Tirana, Albania; 2 Mediterranean and Black Sea Programme in Intervention Epidemiology Training (MediPIET), European Centre for Disease Prevention and Control (ECDC), Stockholm, Sweden; 3 Department of Infectious Disease Control, Institute of Public Health, Tirana, Albania; 4 Department of Veterinary Epidemiology and Biostatistics, Agricultural University of Tirana, Tirana, Albania; University of North Dakota, UNITED STATES OF AMERICA

## Abstract

**Background:**

COVID-19 breakthrough cases raised crucial questions about vaccine effectiveness, implications for public health, and the dynamics of viral transmission. We described vaccine breakthrough infections in Tirana during the Delta (01/07/2021-12/15/2021) and Omicron (16/12/2021–31/03/2022) periods, to better inform mitigation and vaccination strategies.

**Methods:**

We extracted data from 01/04/2021–31/03/2022 (study period) from two systems: the Albanian infectious disease surveillance system, and the national vaccination database. We defined a COVID-19 case as a resident of Tirana who tested (RT-PCR/antigen) positive for SARS-CoV-2 on a respiratory specimen. We defined vaccine breakthrough infection as having a COVID-19 positive specimen collected ≥14 days after the primary series of vaccines (2 doses). We calculated risk of breakthrough infection using the total number of fully vaccinated individuals as denominator and risk ratios (RR) using binomial regression.

**Results:**

During the study period, 23,875 cases were reported in Tirana; 36% (291,445/800,000) individuals were fully vaccinated and among those, 9,156 (3.1%) breakthrough infections were detected. The median time from vaccination to breakthrough infection was 149 (IQR:102–209) days. The risk of breakthrough infection was higher in 0–39-year-olds (RR = 3.01;95%CI = 2.78–3.27) and during the Omicron period (RR = 32; 95%CI = 30.00–34.91). Those receiving Gam-COVID ((RR = 1.64;95%CI = 1.39–2.02), ChAdOx1-S/Vaxzervria (RR = 1.56;95%CI = 1.48–1.65), or BNT162b2-Comirnaty (RR = 1.36;95%CI = 1.31–1.41) had a higher risk of breakthrough infection compared with those vaccinated with CoronaVac.

**Conclusions:**

Incidence of COVID-19 breakthrough infection was significantly higher during the Omicron period and among younger individuals in Tirana. The substantially elevated risk associated with Omicron variant breakthrough infection and the decline in infection protection after a 5-month period, warrants close monitoring and rapid adaptation of vaccination strategies, including promoting booster vaccination to reduce breakthrough infections in the future.

## 1. Introduction

COVID-19 vaccines marked a monumental step forward in the battle against the SARS-CoV-2 virus [1].Vaccination campaigns worldwide offered hope for decreasing the spread of the virus and reducing the severity of infections. Data from randomized clinical trials and observational studies indicated that authorized COVID-19 vaccines, are safe and highly effective for preventing COVID-19–related serious illness, hospitalization, and death, even during the period in which prevalence of the new variants of concern Delta and Omicron increased at the end of 2021 [2–4]. However, the landscape of this pandemic also evolved, with the occurrence of breakthrough COVID-19 infections [5–7].

The first case of SARS CoV-2 infection in Tirana, Albania, was detected on March 8, 2020, reaching a cumulative incidence of 7215 cases per 100,000 with a cumulative mortality of 114 deaths/100,000 population by the end of 2021. COVID-19 vaccination in Tirana started on January 11, 2021, with priority initially given to health care and social workers, long term facilities residents and continued with educators until the launch of a campaign targeting the general population by age group by March 28, 2021. The monovalent vaccines used were the BNT162b2 COMIRNATY (Pfizer-BioNTech), ChAdOx1-S/ Vaxzervria/ Covishield (AstraZeneca/), Gam-COVID-Vac (Sputnik V) and CoronaVac (Sinovac Biotech). Amidst the nationwide administration of the four COVID-19 vaccines in Albania, a diverse cohort also received the mRNA-1273 (Moderna) vaccine abroad. At the beginning of 2022, vaccination with booster doses was implemented, but booster vaccination with bivalent vaccine Pfizer-BioNTech BA.4–5 started in mid-November 2022.

While COVID-19 vaccines prevented or attenuated the severity of SARS-CoV-2 infection, little was known about breakthrough infections and the factors associated with those [5,6]. Breakthrough infections, defined as the occurrence of COVID-19 among individuals who have been fully vaccinated, raised crucial questions about vaccine effectiveness, implications for public health, and the dynamics of viral transmission [5]). We aimed to describe the characteristics and estimate incidence of SARS-CoV-2 breakthrough infections and investigate the association between SARS-CoV-2 variants and breakthrough infections in Tirana during the Delta and Omicron periods to better inform mitigation and vaccination strategies.

## 2. Materials and methods

### 2.1 Study design and population

This retrospective cohort study was conducted across Tirana, Kamez, and Vore municipalities, forming part of the Tirana public health area within the jurisdiction of the Public Health Unit and included all COVID-19 cases reported between April 1, 2021, and March 31, 2022. The three municipalities include a total population of 800,000 inhabitants. We collected data from April 1, 2021, and March 31, 2022. We excluded individuals that were not vaccinated in Albania (vaccinated with mRNA-1273 and Ad26.COV2.S).

### 2.2 Definitions

A COVID-19 case was identified as an individual residing in Tirana whose respiratory specimen tested positive for SARS-CoV-2 RNA or antigen test. A COVID-19 vaccine breakthrough infection case was defined as a COVID-19 case tested positive at least 14 days after completing the primary series of an authorized SARS-CoV-2 vaccine (2 doses-fully vaccinated). Partially vaccinated were those vaccinated with at least one dose but have not completed the vaccine series (2 doses) or those vaccinated within 0–13 days after completing the primary vaccination series. We considered as unvaccinated individuals those who had not received any COVID-19 vaccine. A severe vaccine breakthrough COVID-19 case was a breakthrough case requiring hospitalization at least 14 days after concluding the primary series of an authorized SARS-CoV-2 vaccine. Based on the National Institute of Public Health’s laboratory surveillance reports, we classified specific timeframes for different variants, defining i) the Alpha variant phase as spanning from April 2021 to the end of June 2021, ii) the Delta variant phase between 1 July and mid- December 2021 and iii) the Omicron variant phase as occurring from mid-December 2021 to March 2022.

### 2.3 Data sources

We used interconnected data from two systems: the web-based infectious disease surveillance system (SISI), and the national vaccination system. The web-based infectious disease surveillance system (SISI) constitutes the foundation for collecting and managing crucial health data and functions as a dynamic surveillance platform, integrating diverse data sources from hospitals, clinics, laboratories, local public health directories, and individual reports. Those data include among other, patient demographics, symptoms, laboratory test results, and geographic locations. The main objective of the system was to detect unusual patterns in disease occurrences, enabling early warnings about potential outbreaks or epidemics, and informing rapid response measures. The system operated in real-time, allowing for immediate data input and analysis and ensured confidentiality and security of sensitive health data. The national vaccination system recorded demographic information, vaccination status, vaccination history, including doses administered, dates, types of vaccines, and any adverse reactions for everyone.

### 2.4 Statistical analysis

We linked COVID-19 cases from April 1, 2021 to March 31, 2022, with their respective vaccination status, by using a common identifier (ID). We identified and removed, duplicate cases in the two systems through ID or by using three variables such as name, surname, and date of birth. For all COVID-19 cases without vaccination status recorded in the national vaccination system, we obtained their status from their family doctors’ records. Individuals who experienced multiple infections had their previous infection status excluded from the study, with the analysis including only the newly acquired infections. To calculate the monthly incidence of breakthrough infection, we used as denominators aggregated data of individuals vaccinated with two doses recorded in daily bulletins.

We described the characteristics of breakthrough infections occurring in fully vaccinated individuals during the study period and computed the median time interval (interquartile range (IQR)) since vaccination for breakthrough infections. We compared breakthrough and non-breakthrough infection cases via Pearson χ2 test and we calculated risk ratios with 95% confidence intervals (95%CI) to examine potential associations. We performed the analyses using R statistical software version 4.2.3.

### 2.5 Ethical considerations

Based on the country law on communicable diseases No.15/2016 where all studies related to surveillance can be performed using existing surveillance system, the Institute of Public Health has determined that this study is exempted from further ethical review. The described activities as per study protocol are limited to public health surveillance which meets the criteria for routine public health surveillance as per communicable diseases law Nr.15/2016. Furthermore, obtaining informed consent from patients is not required under public health surveillance.

## 3. Results

Between April 2021 and March 2022, 36% (291,445/800,000) of residents were fully vaccinated in Tirana municipalities (Tirana, Kamez, Vore). During this period, 23,875 1individuals tested positive for SARS-CoV-2 (Table 1).

**Table 1:** Characteristics of individuals with a positive SARS-CoV-2 test in Tirana, April 2021- March 2022 (N = 23,875).

Characteristics	Categories	No Breakthrough				Breakthrough		p-value
		Unvaccinated*		Partially vaccinated†		Fully vaccinated		
		n	%	N	%	n	%	
		(N = 10,367)		(N = 4,352)		(N = 9,156)		
Age Group	0-39	6363	61	2699	62	3823	42	<0.001
	40-69	3481	34	1494	34	4668	51	
	70+	523	5	158	4	665	7.3	
Gender	Male	4522	44	1877	43	3,725	41	<0.001
Vaccine Type	BNT162b2 Comirnaty	–	–	3598	83	4602	50	<0.001
	ChAdOx1-S	–	–	325	8	1926	21	
	CoronaVac	–	–	394	9	2515	28	
	Gam-COVID-Vac	–	–	30	1	113	1	

*Unvaccinated at the time of infection.

†Vaccinated with at least one dose but have not completed the vaccine series (2 doses) or vaccinated within 0–13 days after completing the primary vaccination series (2 doses).

Among new infections, 10,367 (43%) occurred among unvaccinated residents, 4352 (18%) among those who were partially vaccinated or within 0–13 days after completing the primary vaccination series (2 doses), and 9,156 (39%) among fully vaccinated individuals (≥14 days after completing the second dose), indicating that 3.1% of fully vaccinated individuals developed breakthrough infection. Of breakthrough infection cases, 42% were <40 years old, compared with 62% among no breakthrough infection cases (p-value<0.001) (Table 1). Both breakthrough infection cases and cases among partially vaccinated individuals were more likely to have received BNT162b2 COMIRNATY vaccine (50% vs 83%, respectively; p-value<0.001).

Of breakthrough infections, 95 (1%) were hospitalized (severe cases) (Table 2).

**Table 2:** Monthly confirmed COVID-19 cases and hospitalizations among fully vaccinated individuals, Tirana, April 2021- March 2022.

Month	Population	Breakthrough	IR vaccinated	Hospitalized Breakthrough	IR vaccinated hospitalized
	N	n	(n/N*100000)	n	(n/N*100000)
	(N = *291,445)*	(N = *9,156)*		(N = 95)	
Apr.21	9,353	0	0	0	0
May.21	56,766	3	5	0	0
Jun.21	20,793	0	0	0	0
Jul.21	29,949	25	83	0	0
Aug.21	33,100	458	1,384	0	0
Sep.21	45,521	268	589	0	0
Oct.21	23,947	0	0	0	0
Nov.21	19,547	0	0	0	0
Dec.21	18,376	8	44	0	0
Jan.22	18,332	7,418	40,465	62	338
Feb.22	10,467	708	6,764	30	287
Mar.22	5,294	268	5,062	3	57

The incidence of breakthrough infections remained low during April to June 2021 (Alpha period) but increased during the circulation of the Delta variant in July to September 2022, reaching 1,384 cases per 100,000 population. The highest estimated breakthrough monthly incidence among the vaccinated population was observed in January 2022 (40,465 cases/100,000 population) followed by February 2022 (6,764/100,000 population) and March 2022 (5,062/100,000 population) (Table 2). The median number of days between completing the vaccine series and having a breakthrough infection was 149 (inter-quartile range (IQR): 102–209) days.

The incidence of breakthrough infection was higher among 0–39-year-olds, among those vaccinated with Gam-COVID-Vac vaccine and during the Omicron period (Table 3)**.**

**Table 3:** Characteristics of COVID-19 breakthrough infections among fully vaccinated individuals, April 2021 to March 2022, Tirana, Albania.

Category	Characteristics	Fully vaccinated	No breakthrough		Breakthrough		Incidence	Risk Ratio	95%CI	p-value
		n (N = 291,445)	n (N = 282,289)	%	n (N = 9,156)	%	(n/N*100,000)			
Age group	0-39	93,525	89,702	95.9	3823	4.1	4087	3.01	2.78-3.27	<0.001
	40-69	148,899	144,231	96.9	4668	3.1	3135	2.31	2.13- 2.50	
	**70+**	**49,021**	**48,356**	**98.6**	**665**	**1.4**	**1357**	**ref**	–	
Vaccine type	BNT162b2 Comirnaty	136,920	132,318	96.6	4602	3.4	3361	1.36	1.31-1.41	<0.001
	ChAdOx1-S	49,252	47,326	96.1	1926	3.9	3911	1.56	1.48-1.65	
	Gam-COVID-Vac	2,739	2,626	95.9	113	4.1	4126	1.64	1.39-2.02	
	**CoronaVac**	**102,534**	**100,019**	**97.5**	**2515**	**2.5**	**2453**	**ref**	**–**	
Variant	Alpha	86,912	86,909	99.997	3	0.003	4	0.00	0.00-0.02	<0.001
	Omicron	34,093	25,691	75.0	8402	24.6	24644	45	41.8-48.5	
	**Delta**	**170,440**	**169,689**	**99.5**	**751**	**0.4**	**441**	**Ref.**	**–**	

The risk of breakthrough infection among fully vaccinated individuals increased with decreasing age, with the risk being twice higher in 40–69 year-olds and three times higher in 0–39-year-olds, compared with the 70 + year-olds (Table 3). Individuals infected during the Omicron period (RR = 45) were more likely to develop breakthrough infection, when compared with those infected during the Delta period (Table 3). The risks among those who received Gam-COVID-Vac (RR = 1.64), ChAdOx1-S/Vaxzervria (RR = 1.56) and BNT162b2 COMIRNATY (RR = 1.36) were higher compared with those receiving CoronaVac. However, when we stratified by variant, compared with those receiving CoronaVac, the risk among those who received BNT162b2 COMIRNATY was higher during the Alpha period, but lower during the Omicron and Delta periods (Table 4). During the Delta period, the risks among those who received ChAdOx1-S/Vaxzervria and Gam-COVID-Vac were also lower compared with those receiving CoronaVac.

**Table 4:** Incidence of COVID-19 breakthrough infections among fully vaccinated individuals by vaccine type and variant, April 2021 to March 2022, Tirana, Albania.

Variant		Category	Fully vaccinated	Breakthrough		Incidence (n/N*100,000)	Risk ratio	95%CI	p-value
			N	n	(%)				
**Alpha**	Vaccine	BNT162b2 Comirnaty	8048	2	0.02	25	17	1.5-183	0.008
	type	ChAdOx1-S	11435	0	0	0	0	0-NA	
		Gam-COVID-Vac	697	0	0	0	0	0-NA	
		**CoronaVac**	**66732**	**1**	**0.00**	**1.5**	**Ref.**	**–**	
**Omicron**	Vaccine	BNT162b2 Comirnaty	32130	4443	13.8	13828	0.13	0.13-0.14	<0.001
	type	ChAdOx1-S	0	1810	–	–	–	–	
		Gam-COVID-Vac	0	105	–	–	–	–	
		**CoronaVac**	**1963**	**2044**	**104.1**	**104126**	**Ref.**	**–**	
**Delta**	Vaccine	BNT162b2 Comirnaty	96742	157	0.2	162	0.12	0.04-0.05	<0.001
	type	ChAdOx1-S	37817	116	0.3	307	0.22	0.18-0.23	
		Gam-COVID-Vac	2042	8	0.4	392	0.28	0.06-0.35	
		**CoronaVac**	**33839**	**470**	**1.4**	**1389**	**Ref.**	**–**	

## 4. Discussion

The study indicated that breakthrough infections among vaccinated individuals increased substantially in Tirana towards the end of 2021 and remained high in the first three months of 2022, with the risk of breakthrough infection increasing significantly during the Omicron period compared with the Delta or Alpha periods. In our study, 3.1% of fully vaccinated individuals developed breakthrough infection, slightly lower compared with two other studies that reported rates of breakthrough infection at 4% [7} and 7.5% [8}. Several studies globally have reported an increase in breakthrough infections during the period of the Omicron variant circulation, resulting in global concerns regarding the transmissibility of this variant and its potential impact on vaccine efficacy [6–12]. The increased transmissibility of the Omicron variant may be attributed to its higher viral load, potentially leading to reduced vaccine-induced protection from infection. Additionally, the administration of booster doses started in December 2021 in Tirana, with the use of bivalent vaccines introduced later. Consequently, only a small proportion of individuals were vaccinated with booster doses, and many individuals had received their last vaccine dose a long time since vaccination at that period. The median duration between completing the vaccine series and having a breakthrough infection was reduced to 5 months in our study, suggesting a quick decline in infection protection after vaccination. Other studies reported similarly that infection protection was more likely to wane 6 months after the second dose [13,14]. Therefore, factors such as an extended duration since vaccination leading to waning immunity combined with the emergence of the new variant likely contributed to the elevated number of breakthrough infections at that time. The above underscore the importance of close monitoring and rapid adaptation of vaccination strategies, including the administration of booster doses to enhance vaccine-induced immunity.

Our analysis indicated that younger vaccinated individuals had significantly higher risk of contracting the virus and developing breakthrough infections compared with older age groups. These results may be indicative of variations in behaviors related to exposure and adherence to non-pharmaceutical interventions of children and younger adults, such as mask-wearing and social distancing, throughout the study period [15].

The differential risks of breakthrough infection observed among individuals vaccinated with different COVID-19 vaccines may suggest varying levels of effectiveness conferred by each vaccine against infection. We observed differences in those risks when we stratified by variant period, suggesting that COVID-19 variant is a modifier of the effect of vaccine type on breakthrough infection. Similar to our study, a few other studies reported varying incidence rates of breakthrough infections across different vaccine types [13,16]. However, many other studies reported similar vaccine effectiveness of the most-studied COVID-19 vaccines, with most reporting consistently high (>90%) protection against serious clinical outcomes like hospitalizations and deaths, regardless of variant [17,18]. In our study, we could not adjust for several potential confounders that may explain the varying incidence rates of breakthrough infections across different vaccine types and variants.

### 4.1 Limitations

This study had some limitations. Firstly, our study used two distinct data sources, that relied on routinely updated healthcare services data with potential variations in quality; vaccination status was ascertained from the national vaccination dataset, while breakthrough infection status was extracted from the SISI surveillance database. The use of the two differing sources of information could have led to some misclassification. Insufficient staff during the pandemic, a high volume of tasks and complexities in integrating and linking data from divergent sources led to delays in updating vaccination status promptly within the vaccination system, resulting in incomplete information in the vaccination records for numerous individuals. To minimise missing values, we obtained vaccination status from family doctors’ records for COVID-19 cases who did not have their vaccination status recorded in the national vaccination dataset. The SISI surveillance dataset, where the outcome status was recorded, was comprehensive, although some under-reporting cannot be excluded. Our findings align with those of previous studies, providing support for the reliability of the data. Secondly, due to the lack of individual data for all fully vaccinated individuals, we had to rely on aggregated data for part of the analysis, and we could not consider indications for receiving a different type of vaccines or vaccine availability. This limitation prevented us from adjusting for other variables and estimating adjusted measures of effect.

## 5. Conclusions

In conclusion, our analysis indicated that breakthrough infections were more prevalent during the Omicron variant period. The different breakthrough infection rates by vaccine type, emphasize the importance of accounting for vaccine types in public health decision-making. The substantially elevated risk associated with Omicron variant breakthrough infection and the decline in infection protection after a 5-month period, warrants close monitoring and rapid adaptation of vaccination strategies. To mitigate future breakthrough infections, we advocate for the promotion of booster vaccination strategies to enhance overall vaccine effectiveness and provide broader protection against emerging variants of concern. Additionally, our findings emphasize the necessity for ongoing vaccine effectiveness studies to effectively address the dynamic and evolving landscape of SARS-CoV-2 variants.
